# Brain Resilience: The Effect of White Matter Disease on Brain Networks in Cognitively Normal Older Adults

**DOI:** 10.1093/geroni/igaa057.3372

**Published:** 2020-12-16

**Authors:** Blake Neyland, Christina Hugenschmidt, Samuel Lockhart, Laura Baker, Suzanne Craft, Paul Laurienti, Stephen Kritchevsky

**Affiliations:** Wake Forest School of Medicine, Winston-Salem, North Carolina, United States

## Abstract

Brain pathologies are increasingly understood to confer mobility risk, but the malleability of functional brain networks may be a mechanism for mobility reserve. In particular, white matter hyperintensities (WMH) are strongly associated with mobility and alter functional network connectivity. To assess the potential role of brain networks as a mechanism of mobility reserve, 116 participants with MRI from the Brain Networks and Mobility Function (B-NET) were categorized into 4 groups based on median splits of SPPB scores and WMH burden: Expected Healthy (EH: low WMH, SPPB>10, N=45), Expected Impaired (EI: high WMH, SPPB10, N=24), Unexpected Impaired (EI: low WMH, SPPB<10, N=10) and Unexpected Unhealthy (UH: low WMH, SPPB<10, N=37). Functional brain networks were calculated using graph theory methods and white matter hyperintensities were quantified with the Lesion Segmentation Toolbox (LST) in SPM12. Somatomotor cortex community structure (SMC-CS) was similar between UH and EH with both having higher consistency than EI and UI. However, UH displayed a unique increase in second-order connections between the motor cortex and the anterior cingulate. It is possible that this increase in connections is a signal of higher reserve or resilience in UH participants and may indicate a mechanism of compensation in regards to mobility function and advanced WMH burden. These data suggest functional brain networks may be a mechanism for mobility resilience in older adults at mobility risk due to WMH burden.

